# An elderly advanced non-small cell lung cancer patient harboring rare epidermal growth factor receptor mutations L861R benefited from afatinib: A case report

**DOI:** 10.1097/MD.0000000000027614

**Published:** 2021-11-12

**Authors:** Fanjie Qu, Shuang Wu, Huacheng Dong, Xin Yan

**Affiliations:** Department of Oncology, Dalian Third People′s Hospital, Dalian, China.

**Keywords:** advanced non-small cell lung cancer, afatinib, EGFR mutations L861R, rare epidermal growth factor receptor mutations, tyrosine kinase inhibitors

## Abstract

**Rationale:**

Tyrosine kinase inhibitors (TKIs) have significant efficacy in patients with advanced non-small cell lung cancer (NSCLC) with activating epidermal growth factor receptor (EGFR) mutations. No clear evidence exists that EGFR-L861R is sensitive to TKIs, and the best treatment for NSCLC patients with EGFR-L861R mutation is undetermined.

**Patient concerns:**

We report the characteristics, efficacy, and adverse events of a patient harboring rare EGFR mutations L861R treated with afatinib, and summarize the currently available evidence and ongoing clinical trials regarding it.

**Diagnosis:**

The patient was diagnosed with advanced lung cancer that had progressed after previous osimertinib drug therapy, based on the clinical course and imaging findings.

**Interventions:**

The patient underwent genetic testing, and next-generation sequencing detected rare EGFR mutations L861R in the plasma (mutation abundance 8.1%). The patient was then administered afatinib at 30 mg quaque die combined with bevacizumab at 300 mg every 2 weeks.

**Outcomes:**

After 1 month of treatment, the patient achieved a quick response, and symptoms improved significantly. Repeat evaluation imaging demonstrated that the lesions in the lung and brain were significantly smaller and evaluation showed partial remission. However, despite showing an initial response, the patient presented with behavioral abnormalities, headaches, and sudden confusion after 2 months, and subsequently appeared coma. The family elected to forgo further therapy due to the patient's age and enrolled in hospice care, passing 14 months after the initial diagnosis.

**Lesson:**

EGFR-L861R mutation could help predict the sensitivity of patients with advanced NSCLC to TKIs.

## 1 Introduction

Molecular targeted therapy has successfully ushered in a new era of precision therapy for advanced non-small cell lung cancer (NSCLC). The discovery of epidermal growth factor receptor (EGFR) mutations significantly changed the treatment paradigm of patients with EGFR-mutant NSCLC. In these patients, the first-line treatment of choice is first- to third-generation EGFR-tyrosine kinase inhibitors (EGFR-TKIs). EGFR mutations were mainly found between exon 18 to 21 in the coding region of tyrosine kinase, in which 46% of the ELREA amino acid sequence was missing in exon 19, and 35% to 45% of the L858R mutation in exon 21.^[1,2]^ These 2 mutations are the types of mutations that are sensitive to small-molecule TKIs, and the clinical response rate of TKIs in NSCLC patients with sensitive mutations is 62% to 83%.^[3–5]^ Approximately 10% of patients with EGFR mutations carry rare mutations.^[6]^ There is little data on the sensitivity of these tumors to EGFR inhibitors with rare EGFR mutations. No clear evidence exists that EGFR-L861R is sensitive to TKIs, and the best treatment for NSCLC patients with EGFR-L861R mutation is undetermined.

## 2 Case report

**Figure 1:**
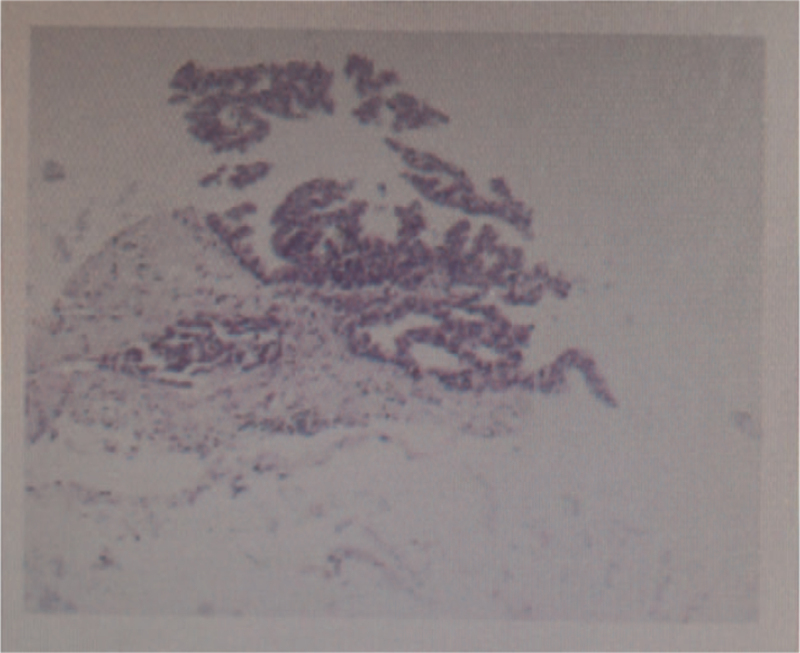
Hemotaxylin and eosin staining results showed adenocarcinoma (×200).

An 84-year-old non-smoking woman was admitted to the hospital in June 2020 with a 3-month cough and shortness of breath. Computed tomography showed mass shadows in the right hilum, atelectasis in the middle lobe of the right lung, and multiple metastases in both the lungs and mediastinum lymph nodes. Her Karnofsky Performance Score was 20. Magnetic resonance imaging revealed multiple brain metastases. An interventional radiology-guided core biopsy of the primary lesion revealed a poorly differentiated adenocarcinoma, and hematoxylin and eosin staining of the lung lesion revealed epithelioid tumor cells with a glandular tubular arrangement (Fig. 1), and immunohistochemistry revealed CK(+), TTF-1(+), and napsin-a(+), which indicated primary lung adenocarcinoma. The patient was diagnosed with stage IV lung adenocarcinoma.

**Figure 2:**
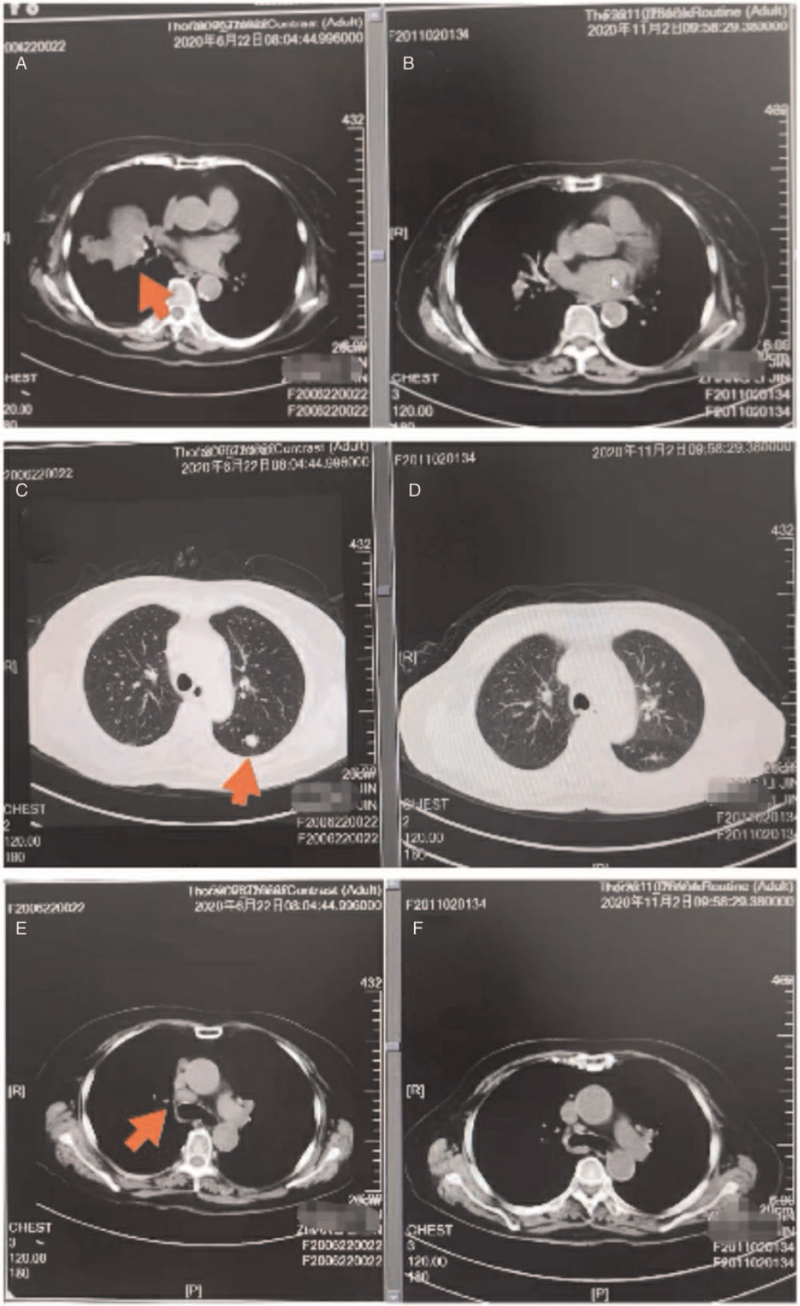
The pulmonary computed tomography (CT) of different time showed mass shadows in the right hilum, atelectasis in the middle lobe of the right lung, and multiple metastases in both lungs and mediastinum lymph nodes. (A,C,E) (2020-06-22) before “osimertinib” treatment. (B,D,F) (2020-11-02) after “osimertinib” treatment was administered for 4 months.

**Figure 3:**
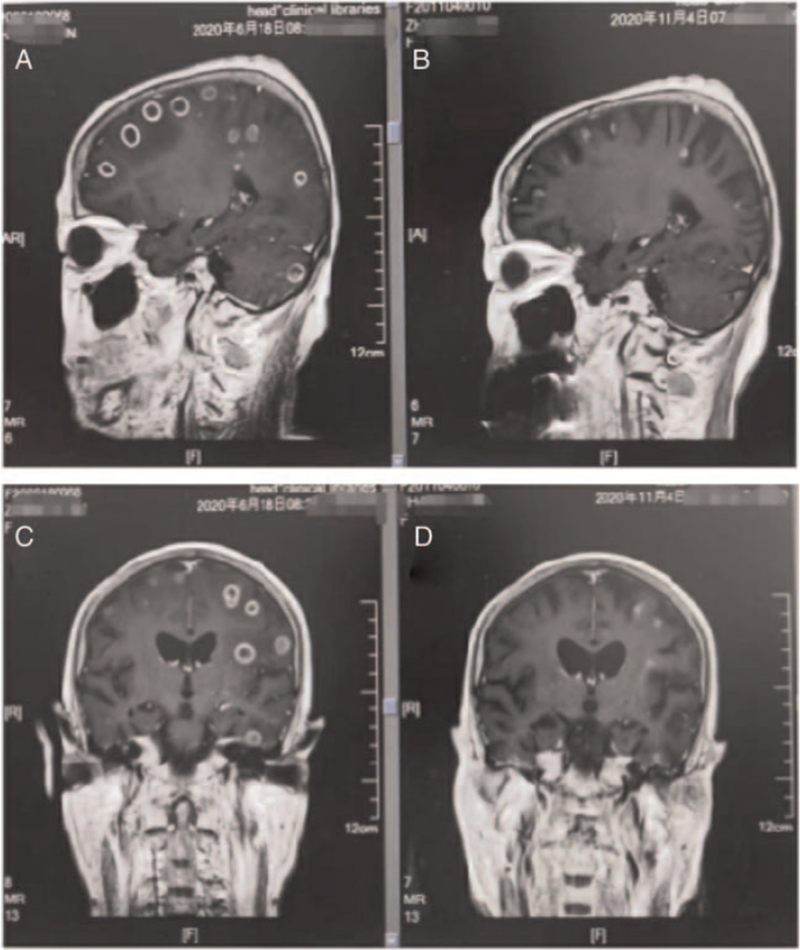
The brain magnetic resonance imaging (MRI) of different time showed multiple brain metastasis. (A,C) (2020-06-18) before “osimertinib” treatment. (B,D) (2020-11-04) after “osimertinib” treatment was administered for 4 months.

Subsequently, next-generation sequencing detected the EGFR mutation L858R in lung biopsy tissues (mutation abundance, 34.6%). The patient was administered first-line treatment with osimertinib at 80 mg quaque die (QD) from July 2020. The clinical discomfort symptoms were quickly relieved after a month of treatment, and re-examination showed considerable shrinkage of the pulmonary nodules and brain metastasis nodules; as first-line therapy, osimertinib was prescribed for about 10 months and the best therapeutic evaluation was partial remission (Figs. 2 and 3). The main adverse events were grade 2 diarrhea and skin rash.

**Figure 4:**
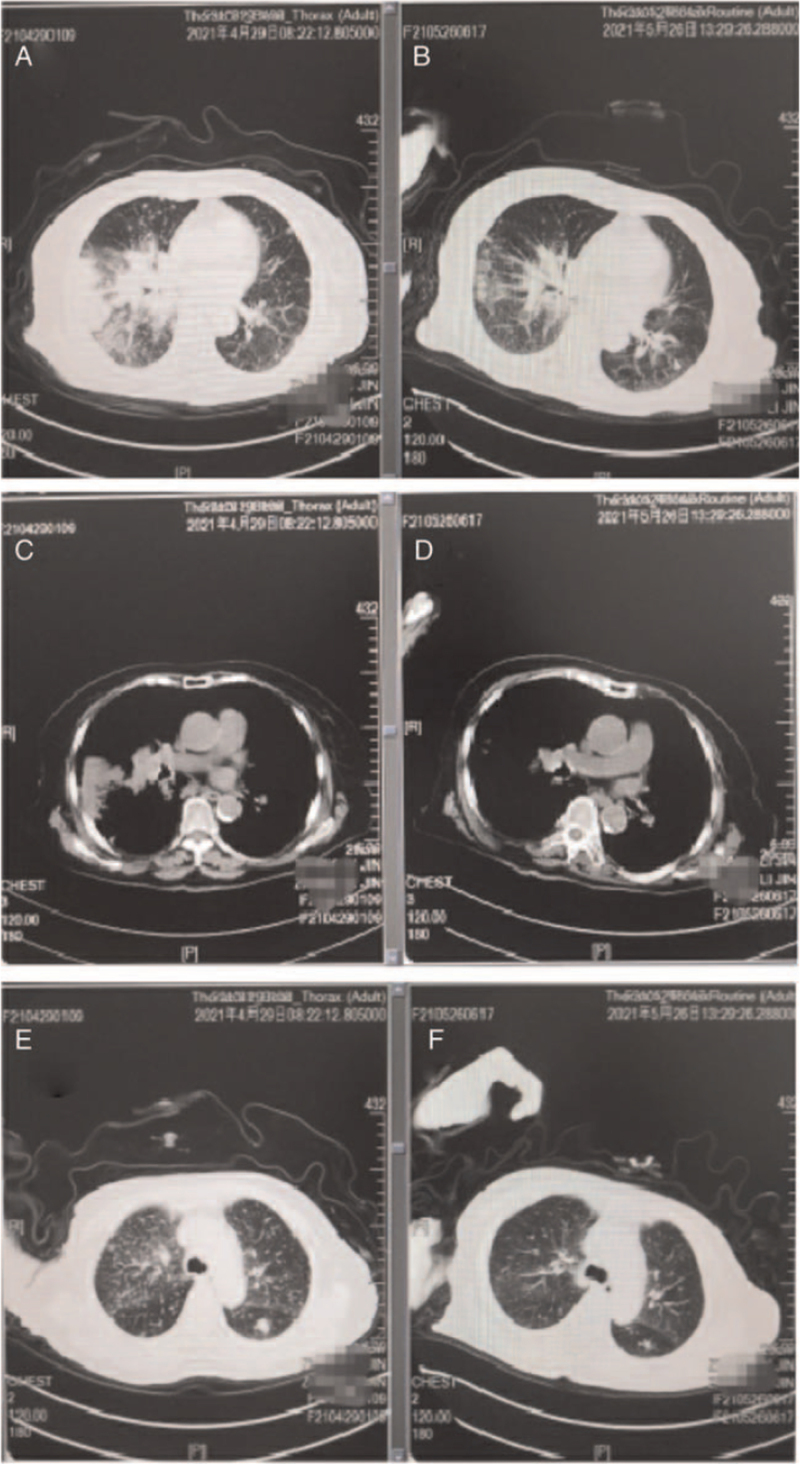
The pulmonary computed tomography (CT) of different time showed mass shadows in the right hilum, atelectasis in the middle lobe of the right lung, and multiple metastases in both lungs. (A,C,E) (2021-04-29) before “afatinib plus bevacizumab” treatment. (B,D,F) (2020-05-26) after “afatinib plus bevacizumab” treatment was administered for 20 days.

**Figure 5:**
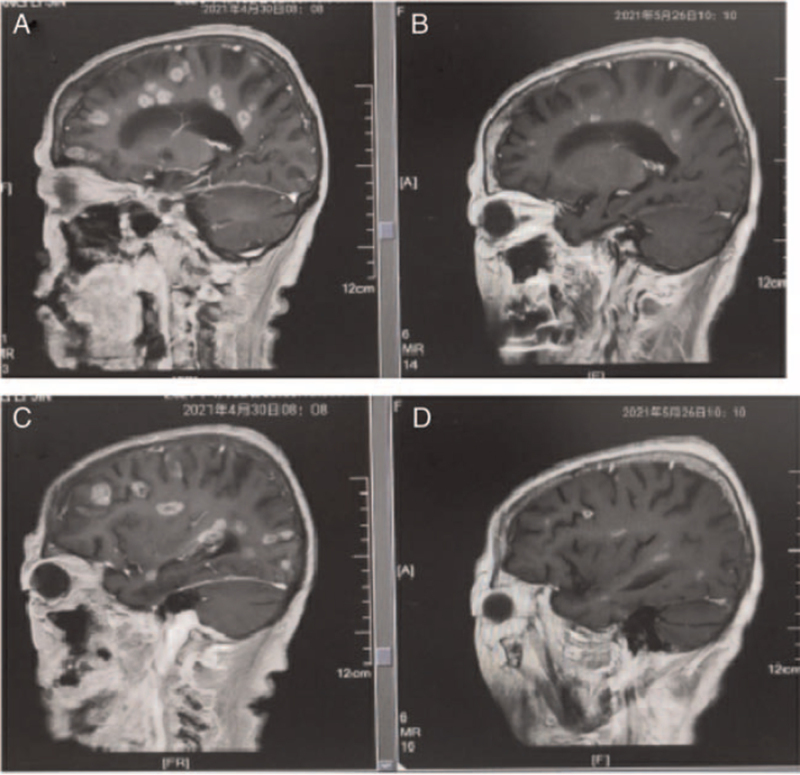
The brain magnetic resonance imaging (MRI) of different time showed multiple brain metastasis. (A,C) (2021-04-30) before “afatinib plus bevacizumab” treatment. (B,D) (2020-05-26) after “afatinib plus bevacizumab” treatment was administered for 20 days.

However, the disease progressed after 10 months, and the patient reappeared with cough and shortness of breath, accompanied by dizziness and nausea, and weight loss in April 2021. Computed tomography and magnetic resonance imaging showed progression of lung lesions and brain metastases. The patient underwent genetic testing (Illumina sequencing platform) in April 2021, and next generation sequencing detected rare EGFR mutations L861R in plasma (mutation abundance 8.1%). The patient was then administered afatinib at 30 mg QD combined with bevacizumab at 300 mg every 2 weeks as second-line treatment from May 2021; after 1 month of treatment, the patient achieved a quick response, and symptoms such as cough and asthma, nausea, and poor appetite improved significantly, and weight gained. Repeat evaluation imaging demonstrated that the lesions in the lung and brain were significantly smaller and evaluation showed partial remission after 20 days (Figs. 4 and 5). In addition, the toxicities experienced by the patient were mainly grade 1 hand–foot skin reactions and hypertension, which were well managed.

However, despite showing an initial response, the patient presented with behavioral abnormalities, headaches, and sudden confusion after 2 months, and subsequently appeared coma. She displayed no fever, cough, or dyspnea. The family elected to forgo further therapy due to the patient's age and enrolled in hospice care, passing 14 months after the initial diagnosis.

In this case report, the patients’ responses to all drugs were according to RECIST criteria and adverse events were as per CTCAE criteria.

All procedures performed in studies involving human participants were in accordance with the ethical standards of the institutional and/or national research committee(s) and with the Helsinki Declaration (as revised in 2013). Written informed consent was obtained from all the patients.

## 3 Discussion

The discovery of EGFR mutations significantly changed the treatment paradigm of patients with EGFR-mutant NSCLC. In these patients, the first-line treatment of choice is first- to third-generation EGFR-TKIs. A phase 3 trial^[7,8]^ compared first-line osimertinib with other EGFR-TKIs in patients with EGFR-sensitive mutations advanced NSCLC in the FLAURA trial. The trial showed longer progression free survival (PFS) and longer overall survival (OS) with osimertinib than with the comparator EGFR-TKIs.

In the recently updated FLAURA phase 3 study of 556 previously untreated NSCLC patients harboring EGFR exon 19 deletion or L858R mutation, osimertinib showed superiority over the standard EGFR-TKIs gefitinib or erlotinib in median OS (38.6 vs 31.8 months) and a similar safety profile (adverse events of grade 3 or higher 42% vs 47%).^[9]^ Subsequently, it was also approved as a first-line treatment for metastatic NSCLC patients harboring EGFR exon 19 deletions or L858R mutations.

Osimertinib, an oral, third-generation TKI with the ability to penetrate the BBB,^[10]^ has been approved for the treatment of metastatic NSCLC patients with EGFR mutation.

Therefore, when the EGFR 21del L858R mutation was detected in the patient's lung tissue samples, we decided to treat the patient with osimertinib at 80 mg QD as first-line treatment, which led to a PFS of 10 months. However, the disease progressed after 10 months. Molecular testing of the plasma detected rare EGFR mutations L861R. At present, the efficacy TKI for treating patients with NSCLC who carry uncommon EGFR mutations have not been determined. Approximately 10% of patients with EGFR mutations carry rare mutations. There is little data on the sensitivity of these tumors with rare EGFR mutations to EGFR inhibitors, and the study showed that L861 mutations in exon 21 were uncommon EGFR mutations, accounting for approximately 2% of EGFR mutations, and the effective rate of TKI treatment was 41/60%. PFS or OS is better than the wild type, although it is slightly worse than the common mutations in EGFR.^[11]^

Afatinib was first approved by the U.S. Food and Drug Administration on July 13, 2013, as a first-line treatment for NSCLC with an EGFR 19 exon deletion mutation or exon 21 L858R mutation.^[12,13]^ Recently, the clinical activity of afatinib in NSCLC patients with rare EGFR mutations has been reported, and Yang et al^[6,14]^ reported the results of LUX-Lung2, Lux-LunG3, and lux-Lung6 clinical trials of afatinib in patients with advanced NSCLC with non-classical mutations, which showed good reactivity to afatinib. Based on these findings, the Food and Drug Administration approved afatinib for the treatment of metastatic NSCLC with non-classical EGFR mutations.

To achieve a better disease response rate and longer PFS, exploration of drug combinations continues to emerge. Studies have indicated that bevacizumab combined with targeted therapy can inhibit tumor progression and prolong the survival time of NSCLC patients, and vascular endothelial growth factor is correlated with the EGFR pathway, stage II (JO25567),^[15]^ and stage III (NEJ026),^[16]^ and clinical trials have shown that bevacizumab combined with EGFR-TKIs can prolong the PFS time of patients and delay the drug resistance time of EGFR-TKIs.

In a multicenter, one-arm, phase II trial (ABC study),^[17]^ subjects were treated with a combination of afatinib and bevacizumab. The results showed that the combination of afatinib and bevacizumab was effective in the treatment of lung cancer, with no treatment-related death, interstitial lung disease, or severe bleeding associated with bevacizumab.

Therefore, when the rare EGFR 21del L861R mutation was detected in the patient's blood samples, we decided to treat the patient with afatinib 30 mg once daily in combination with bevacizumab every 2 weeks. As described in this case report, afatinib exhibited an encouraging response and achieved a quick response for only 20 days.

An important point to note from this case is that targeted therapy was chosen only because the patient was deemed too weak to undergo radiotherapy or chemotherapy. Based on the rare EGFR L861R (exon 21) mutations detected in the plasma, afatinib (30 mg daily) was administered in combination with bevacizumab at 300 mg every 2 weeks. In conclusion, afatinib treatment achieved an encouraging clinical response in a patient with NSCLC harboring the rare EGFR mutation L861R. To our knowledge, this study provides further clinical evidence for the administration of afatinib for treating NSCLC patients harboring EGFRL861R mutations.

This is a rare case of a patient who initially presented with EGFR mutation L858R, which was treated with osimertinib at 80 mg QD, led to a PFS of 10 months, and was subsequently presented with rare EGFR mutation L858R, which benefited from afatinib. Therefore, where possible, it is advisable to perform more biopsies or obtain plasma for identification purposes in similar cases. Since only 1 patient with advanced lung cancer was observed in this report, the clinical data are very limited and further observation and accumulation of more experience are needed, and further clinical studies will be conducted on the efficacy and safety of this combination regimen. However, when the patient is administered afatinib, it should be pointed out attention must be paid to common adverse reactions such as rash and diarrhea.

## Author contributions

Qu Fanjie, Yan Xin, and Wu Shuang were responsible for collecting data, sorting out data, and writing the paper; Yan Xin was responsible for guiding the writing and participating in the revision of the paper; all authors read and approved the final manuscript.

**Conceptualization:** Xin Yan.

**Data curation:** Fanjie Qu, Shuang Wu, Huacheng Dong, Xin Yan.

**Formal analysis:** Fanjie Qu, Shuang Wu.

**Funding acquisition:** Fanjie Qu.

**Investigation:** Fanjie Qu, Shuang Wu, Huacheng Dong.

**Project administration:** Fanjie Qu, Xin Yan.

**Resources:** Fanjie Qu, Huacheng Dong.

**Writing – original draft:** Fanjie Qu, Shuang Wu.

**Writing – review & editing:** Fanjie Qu, Xin Yan.
